# Serological signatures of declining exposure following intensification of integrated malaria control in two rural Senegalese communities

**DOI:** 10.1371/journal.pone.0179146

**Published:** 2017-06-13

**Authors:** Ronald Perraut, Marie-Louise Varela, Cheikh Loucoubar, Oumy Niass, Awa Sidibé, Adama Tall, Jean-François Trape, Amele Nyedzie Wotodjo, Babacar Mbengue, Cheikh Sokhna, Inès Vigan-Womas, Aissatou Touré, Vincent Richard, Odile Mercereau-Puijalon

**Affiliations:** 1 Institut Pasteur de Dakar, Unité d’Immunologie, Dakar, Sénégal; 2 Institut Pasteur de Dakar, G4 Biostatistiques Bioinformatique et Modélisation, Dakar, Sénégal; 3 Institut Pasteur de Dakar, Unité d’Epidémiologie, Dakar, Sénégal; 4 Institut de Recherche pour le Développement (IRD), URMITE, Dakar, Sénégal; 5 Institut Pasteur de Dakar, Unité d’Immunogénétique, Dakar, Sénégal; 6 Institut Pasteur de Madagascar, Unité d’Immunologie des Maladies Infectieuses, Antanarivo, Madagascar; 7 Institut Pasteur, Department of Parasitology and Insect Vectors, 25 Rue du Dr Roux, Paris, France; Université Pierre et Marie Curie, FRANCE

## Abstract

Recent control scale-up has reduced malaria in many areas but new tools are needed to monitor further progress, including indicators of decreasing exposure to parasite infection. Although serology is considered a promising approach in this regard, the serological impact of control interventions has been so far studied using indirect quantification of exposure. Cohort surveys concomitantly recording entomological and malariometric indices have been conducted in two Senegalese settings where supervised control intensification implemented in 2006 shifted malaria from historically holoendemic in Dielmo and mesoendemic in Ndiop to hypoendemic in both settings by 2013. We analyse here serological signatures of declining transmission using archived blood samples. Responses against ten pre-erythrocytic and erythrocytic antigens from *Plasmodium falciparum* and *P*. *malariae* alongside an *Anopheles gambiae* salivary gland antigen were analysed. Cross-sectional surveys conducted before (2002) and after (2013) control intensification showed a major impact of control intensification in both settings. The age-associated prevalence, magnitude and breadth of the IgG responses to all antigens were village-specific in 2002. In 2013, remarkably similar patterns were observed in both villages, with marginal responses against all parasite antigens in the 0-5y children and reduced responses in all previously seropositive age groups. Waning of humoral responses of individuals who were immune at the time of control intensification was studied from 2006 to 2013 using yearly samplings. Longitudinal data were analysed using the Cochran-Armittage trend test and an age-related reversible catalytic conversion model. This showed that the antigen-specific antibody declines were more rapid in older children than adults. There was a strong association of antibody decline with the declining entomological inoculation rate. We thus identified serological markers of declining exposure to malaria parasites that should help future monitoring of progress towards malaria elimination.

## Introduction

Intensification of integrated control interventions have considerably reduced the global burden of malaria [1]. There remain however multiple challenges to reaching elimination, including developing indicators of declining exposure to infection to monitor progress[2]. Indeed, collection of age-associated case distribution, which varies by transmission intensity, becomes logistically challenging in the context of declining malaria and moreover overlooks the asymptomatic infection reservoir[3]. Screening campaigns using sensitive molecular assays only provide a snapshot record of infection. Entomological methods lack sensitivity when transmission is low. Serology provides a signature of exposure to parasites overcoming many of these caveats[4]. A large number of parasite antigens elicit robust humoral responses and numerous assays have been used, based on parasite preparations[5–8]recombinant proteins or peptides in monoplex or mutiplex formats [8–19]. Seroprevalence and antibody levels to *P*. *falciparum* antigens are associated with transmission intensity but the relationship is non-linear and antigen- and age-dependent [4, 9, 12, 19–23]. However, these conclusions stem from comparison of sites with differing socioeconomic and/or climatic conditions, being either located distantly [21, 23–26] or at different altitudes [11, 12, 19, 27–29], from short-term follow up [7, 9, 13, 21, 22, 30–34] or from studies focussing on specific age groups [9, 21, 24, 31, 32]. Decreased antibody responses are observed several years after successful control intervention(s) [5, 7, 8, 35–39]. Some antibody responses seem short-lived [9, 13, 26], while others persist after extended periods of interrupted exposure [40, 41]. Analysis of the temporal dynamics of antibody responses in communities after supervised interventions and quantified impact on entomological transmission is lacking.

To gain insight into the consequences of control interventions and transmission decline on the antibody responses, we analyse here two communities living in a rural area of Senegal under the same climatic conditions albeit distinct historical malaria endemicity [42–46]. In both settings, *P*. *falciparum* dominates, but *P*. *malariae* and *P*. *ovale* are endemic as well [42, 47, 48]. High coverage, supervised control interventions have been introduced simultaneously in both communities. In 2006, an artemisinin-based combination therapy (ACT) was introduced as first-line treatment. Long-lasting insecticide-treated nets (LLINs) were provided to each household in July 2008 and renewed in 2011 [46]. The incidence of malaria attacks decreased after 2006 and dropped to low levels after 2008 [46, 47, 49]. Some rebound was observed in 2011, the highest incidence being in the older children and young adults[42, 46]. Importantly, parasite rates dramatically declined a few months after LLIN deployment, and transmission of parasites dropped markedly after LLIN renewal in 2011[46, 48, 49].

We profile here the IgG responses of archived blood samples using a multistage, multispecies approach. We first analyse cross-sectional blood samplings made at the end of the dry season in the years 2002 and 2013and monitor responses to a panel of antigens used in previous studies as inducing high seroprevalence in malaria-exposed communities [10, 14, 34]. We then analyse on a yearly basis the waning of responses to three antigens during the years 2006–2013 in previously immune age groups. This data shows that control intensification induced major changes in the age-stratified antibody profiles, reflecting age-dependent seroreversion of previously immune age groups and minimal responses in young children. The similar response profiles of both communities in 2013 provide serological signatures of efficacious transmission decline in the previous years that should help future monitoring of declining immunity in communities during the transition to elimination.

## Material and methods

### Study sites, procedures and ethics

Dielmo and Ndiop are located 5 km apart in the district of Fatick, Senegal, and exposed to the same climatic environment but distinct historical transmission/endemicity conditions, with perennial and intense transmission in Dielmo situated on the banks of a stream[44, 46, 48], and moderate and highly seasonal in Ndiop[42–45].The long-term observational studies of malaria implemented in these communities uninterruptedly record for more than 20 years the episodes of fever, clinical malaria, malaria infection, parasite and vector species and entomological inoculation rate in both settings. The protocol was approved by the National Senegalese Health Research Ethics Committee. Informed consent was obtained from all participants or their parents or guardians in the presence of an independent witness.

The procedures, similar for both villages, have been described in detail [42, 43, 45, 46]. Briefly, clinical cases were monitored daily by active and passive case detection and parasite rates were measured quaterly. Prompt diagnosis and treatment were provided on site. In October 2003, chloroquine was replaced as first-line treatment of *falciparum* malaria for the amodiaquine /sulfadoxine-pyrimethamine combination itself replaced in May 2006 for the artesunate/ amodiaquine combination. Transmission monitoring included measurement of vector density by human-landing catches. The monthly human biting rate and the proportion of infected mosquitoes identified by ELISA for the *P*. *falciparum* circumsporozoite protein were used to determine the entomological inoculation rate (EIR), i.e. the mean number of infected mosquito bites per person per year[45, 46]. In July 2008, vector control was massively implemented with provision of long-lasting insecticide-treated nets (LLINs) to each household. New LLINs were provided in July 2011 [46].

The protocol includes a monthly systematic and a yearly cross-sectional blood sampling. Samples are frozen and archived. Two cross-sectional samplings, conducted in July 2002 and July 2013 at the end of the dry season (Table 1) as well as monthly capillary samples withdrawn from 2006 to 2013 have been studied here.

**Table 1:** Malaria morbidity, parasite carriage and parasite transmission in Dielmo and Ndiop during the 12 months preceding the cross-sectional surveys.

	Dielmo		Ndiop	
	2002	2013	2002	2013
**Clinical follow up**				
No days of follow-up	91 576	139 475	113 571	130 861
< 7 years	24 252	32 241	32 848	38 512
7–14 years	25 311	33 087	28 254	25 079
15–29 years	17 806	29 955	26 644	32 105
≥ 30 years	24 207	44 192	25 825	35 165
Annual incidence of malaria attacks (100 pers*days)				
overall	0.59	0.04	0.44	0.07
< 7 years	1.69	0.03	0.75	0.06
7–14 years	0.51	0.07	0.66	0.10
15–29 years	0.15	0.07	0.21	0.07
≥ 30 years	0.08	0.02	0.07	0.04
**Parasite carriage in cross sectional studies**	** **	** **	** **	** **
% positive bloodsmears^a^	46.1%^a^	0%	19.8%^a^	0%
% PCR positive samples	N.T	4.6%	N.T	2.9%
**Entomological Inoculation Rate (EIR)**	** **	** **	** **	** **
Cumulative EIR 1^st^ July to 30^th^ June next year^b^	215.5	7.5^c^	171.8	2.5

Asymptomatic carriage (presence of circulating blood stages detected by microscopy in healthy subjects) was detected by examining Giemsa-stained blood smears by microscopy, the microscopic detection level being in our hands 2 parasites/uLblood[48] and in 2013 by nested PCR [50].

^a ^Giemsa-stained thick blood smears examined by microscopy; N.T = not tested

^b ^yearly No infected bites/pers/year—infected bites/pers from 1st July 2001 to 30th June 2002 and 1st July 2012 to 30th June 2013

^c ^Introduction of ACT as first line treatment in Jan 2006, Universal LLINs distribution in July 2008 and LLINs replaced for new ones in July 2011

Blood stages and parasite species were numerated using microscopy[46, 48]. Plasmodium DNA was detected in blood samples by nested, species-specific PCR [50].

### Antigens

The *P*. *falciparum* schizont extract (SE) of the 07/03 Dielmo strain was prepared as described [5, 15, 51, 52]. PF13, the recombinant NTS-DBL1α1 domain encoded by the 3D7/PF13_0003 *P*. *falciparumvar* gene was produced in *Escherichia coli* as reported [53]. PfMSP1p19, the 19 kDa C-terminal domain of the *P*. *falciparum* Merozoite Surface Protein-1 [54], produced using a baculovirus-insect cell expression system, was a kind gift of S. Longacre (Vaximax, Genopole Evry, France). Peptide synthesis and coupling to Bovine Serum Albumin (BSA) were custom made by GenScript HK Inc., Hong Kong, China, or Genecust, France. Purity of each BSA-peptide was estimated >85% by HPLC and mass spectrometry. The sequence of the peptides used [10, 14] is displayed in Table 2.

**Table 2:** Antigens used in this study.

Antigen	Type	Region No^a^	Species	Stages of expression^b^	Peptide sequence or proteindomain^c^^,^^d^	ref
*Pf*CSP	peptide	26	*P*. *falciparum*	sporozoite, intrahepatic	(NANP)_9_NVDPNVDPC	[10]
LSA1_41_	peptide	27	*P*. *falciparum*	intrahepatic	(LAKEKLQEQQSDLEQER)_2_LAKEKEKLQC	[10]
SALSA	peptide	33	*P*. *falciparum*	merozoite	SAEKKDEKEASEQGEESHKKENSQESAC	[10]
GLURP	peptide	29	*P*. *falciparum*	merozoite	EDKNEKGQHEIVEVEEILC	[10]
AMA1	peptide	36	*P*. *falciparum*	sporozoite, merozoite	YKDEIKKEIERESKRIKLNDNDDEGNKKIIAPRIFISDDKDSLKC	[34]
PF13	protein	35	*P*. *falciparum*	sequesteredblood stages	DBL1 domain of PF3D7_1300300	[53]
MSP1p19	protein	34	*P*. *falciparum*	merozoite	C-terminal fragment of MSP1	[54]
*Pm*CSP	peptide	37	*P*. *malariae*	sporozoite, intrahepatic	(NAAG)_10_NDAGC	[34]
gSG6	peptide	38	*A*. *gambiae*	salivary gland	EKVWVDRDNVYCGHLDCTRVATFC	[10,17]
BSA	protein	39	*Bostaurus*	-	-	

^a ^Region number of the fluorescent dye of the magnetic beads

^b^For expression profiles and protein characteristics, see www.plasmodb.org

^c^ The LSA1_41_ peptide is derived from the well-conserved central repeat region of LSA1 (Liver Stage antigen 1) encoded by gene PF3D7_1036400. The SALSA peptide is derived from the well-conserved region encompassing residues 76 to 101 of the merozoite surface protein 4 encoded by gene PF3D7_0207000. The GLURP peptideis derived from a well-conserved repeat region of the glutamate-rich protein encoded by gene PF3D7_1035300. The AMA1 peptide is derived from the well-conserved region encompassing residues 446 to 489 of the apical membrane antigen 1 encoded by gene PF3D7_1133400. The PF13 recombinant protein corresponds to the NTS-DBL1 α domain of the variant Plasmodium Eythrocyte membrane antigen 1 encoded by the *var* gene PF3D7_1300300. The domain is polymorphic. The MSP1p19 protein corresponds to Palo Alto sequence of the highly conserved C-terminal double EGF domain (block 17) of the Merozoite Surface Protein 1 encoded by gene PF3D7_0930300.

^d^ All peptides have an extra C-terminal Cys residue to promote coupling to the carrier BSA.

### ELISA

Indirect ELISA was used to analyse the response against SE in the cross-sectional and longitudinal studies, as well as the longitudinal response against the LSA1_41_ peptide and the recombinant MSP1p19. Procedures were performed using sera diluted 1/200 as described [5, 15, 16]. A pool of sera from immune adults from Dielmo and a pool of European and African non-immune sera were included in each assay as positive and negative controls, respectively. IgG Levels were expressed as OD ratio = OD_sample_ / mean OD_naive pool_. Sera showing an OD ratio >2, corresponding to the mean OD_naive pool_ + 3 SD were considered positive.

### Bead-based multiplex assay

A multiplex assay was used to monitor the response the panel of individual antigens listed in Table 2. Covalent coupling of antigens to carboxylated magnetic Luminex beads and the custom magnetic bead-based Luminex multiplex assay including the antigens listed in Table 2 were done as described [10, 14, 16, 55]. IgG levels were expressed as Mean Fluorescence Intensity (MFI). The background signal against BSA was negligible (around 50–80 for all antigens) and was subtracted from all MFI values (net value). The positivity cut-off was set as above the net MFI + 3 SD of naïve controls.

### Statistical analysis

Categorical variables were compared using the Fisher exact test, continuous variables of antibody responses were analysed using the Kruskal Wallis and the Spearman rank correlation test for non-normally distributed data. Cochrane-t linear and logistic regressions adjusted for age groups or age were used for comparison of categorical and continuous variables. P values <0.05 were considered significant. Longitudinal data were analysed using the Cochran-Armittage trend test. Statistical analyses were performed with R and Statview 5.0^®^ software.

Seroconversion (SCR) and seroreversion (SRR) rates were calculated using an age-specific reversible catalytic conversion model [12]. The model was constructed based on the longitudinal data stratified into pools of 2-year for a given period and for a given antigen.

## Results

In 2002, malaria was typically holoendemic in Dielmo[46] and mesoendemic in Ndiop[42, 44, 45]. Between 2002 and 2013, malaria case incidence decreased by 14.7-fold in Dielmo and 6.3-fold in Ndiop (Table 1). Asymptomatic, microscopically-positive parasite carriage decreased from 46.1% in Dielmo and 19.8% in Ndiop in 2002 to undetectable levels in both settings in 2013. Sub-microscopic infections were detected in 2013 by PCR in 4.6 and 2.9% blood samples from Dielmo and Ndiop, respectively.

Two cross-sectional surveys conducted before the rainy season (July 2002 and July 2013) included 184 and 196 villagers in Dielmo, and 202 and 216 villagers in Ndiop, respectively. In Dielmo and Ndiop, 77 (3.8–80 yo in 2002) and 87 (3.4–68.1 yo in 2002) individuals were commonly present in 2002 and 2013, they were considered independently in their respective age-groups as function of the year. Age distribution and mean age were similar for both surveys in each village and did not differ between the villages (Table 3). The mean number of infected bites per person during the previous 12-month was 215.5 in Dielmo and 171.8 in Ndiop in 2002 (an unusually high transmission was recorded in Ndiop during August-December 2002, contrasting with a mean of 37.1 from 1993 to 2001 and 37.8 from 2003 to 2007). It dropped to 7.5 and 2.5 in 2013 in Dielmo and Ndiop, respectively (Table 1).

**Table 3:** Characteristics of the groups studied in the cross sectional surveys conducted in July 2002 and July 2013.

	Dielmo^c^				Ndiop^c^			
	year 2002^b^		year 2013^b^		year 2002^b^		year 2013^b^	
Studypopulation n =	184		196		202		216	
Sex	No	%	No	%	No	%	No	%
male	86	46.7	73	37.2	93	46.0	75	34.7
female	98	53.3	123	62.8	109	54.0	141	65.3
Age group^a^	No	%	No	%	No	%	No	%
<7 years	19	10.3	19	9.7	29	14.4	27	12.5
[7–14] years	36	19.6	37	18.9	54	26.7	45	20.8
[15–29] years	53	28.8	58	29.6	53	26.2	68	31.5
≥ 30 years	76	41.3	82	41.8	66	32.7	76	35.2
Meanage	28.2		28.9		24.3		25.7	

^a^Age-group at the end of the year

^b ^The intra-village age distribution of individuals did not differ for the 2002 and 2013 surveys: Dielmo P = 0.99; Ndiop P = 0.39 (Chi-square test for each age class and each survey)

^c ^The distribution of individuals studied did not differ between the two villages for each survey, i.e. Dielmo*vs*Ndiop: year 2002 P = 0.14;Dielmo*vs*Ndiop: year 2013 P = 0.53 (Chi-square test for each age class and each year)

### Decreased seroprevalence in all age groups in 2013

We first analysed the cross-sectional blood samplings made in the years 2002 and 2013.A multiplex bead-based approach was used to monitor the responses against individual antigens derived from erythrocytic and exoerythrocytic *P*. *falciparum* stages, *P*. *malariae* exoerythrocytic stages as well as *an A*. *gambiae* salivary gland antigen (Table 2). This panel included peptides and recombinant antigens known to elicit high seroprevalence in these settings [10, 15, 16, 53]. In parallel, we monitored the responses against the complex pool of erythrocyte stage antigens included in SE using ELISA also previously used in these communities [5, 52, 56–58] and providing interesting information in a previous study of immunity decay in Dielmo and Ndiop[5].

From 2002 to 2013, the community seroprevalence decreased in both villages by 26% to 66% depending on the antigen, and this was significant for all antigens (Table 4). Between-village comparison showed higher community seroprevalence for SE, PfCSP, LSA1_41_, GLURP, PF13 and PmCSP in Dielmo in 2002, but no difference in 2013, apart from PmCSP.

**Table 4:** Community seroprevalence for the set of antigens in two crosssectional studies conducted in Dielmo and Ndiop in July 2002 and July 2013.

	Dielmo				Ndiop				Inter-village comparison *P*^c^	
Antigen	seroprevalence		2013 *vs* 2002^a^		seroprevalence		2013 *vs* 2002^a^			
	year 2002	year 2013		*P*^*b*^	year 2002	year 2013		*P*^*b*^	2002	2013
**Schizontextract**	93.4	64.9	-30%	<10^−3^	78.7	56.3	-29%	<10^−3^	<10^−4^	0.085
***Pf*CSP**	70.1	39.8	-43%	<10^−3^	58.9	39.8	-32%	<10^−3^	0.02	0.840
**LSA1**_**41**_	84.2	62.2	-26%	<10^−3^	72.8	52.3	-28%	<10^−3^	0.006	0.046
**SALSA**	53.8	29.1	-46%	<10^−3^	51.9	30.6	-41%	<10^−3^	0.759	0.748
**GLURP**	78.3	50.5	-35%	<10^−3^	67.3	48.6	-28%	<10^−3^	0.017	0.767
**AMA1**	53.8	18.4	-66%	<10^−3^	50.9	19.4	-62%	<10^−3^	0.611	0.802
**PF13**	87.5	44.9	-49%	<10^−3^	64.4	43.1	-33%	<10^−3^	<10^−4^	0.765
**MSP1p19**	79.3	57.7	-27%	<10^−3^	74.3	52.3	-30%	<10^−3^	0.278	0.321
***Pm*CSP**	79.3	49.0	-38%	<10^−3^	42.1	29.6	-30%	0.01	<10^−4^	<10^−4^
**gSG6**	26.6	17.9	-33%	0.04	35.1	16.6	-53%	<10^−3^	0.557	0.066

^a ^Percent seroprevalence observed in 2002 no longer observed in 2013

^b ^Comparison of antigen-specific seroprevalence in 2002 vs 2013 (Chi-square test)

^c^ Between-village comparison of antigen-specific community seroprevalence observed at each cross-sectional survey (i.eDielmo*vs*Ndiop in July 2002, Dielmo*vs*Ndiop in July 2013) (Chi-square test)

**Fig 1:**
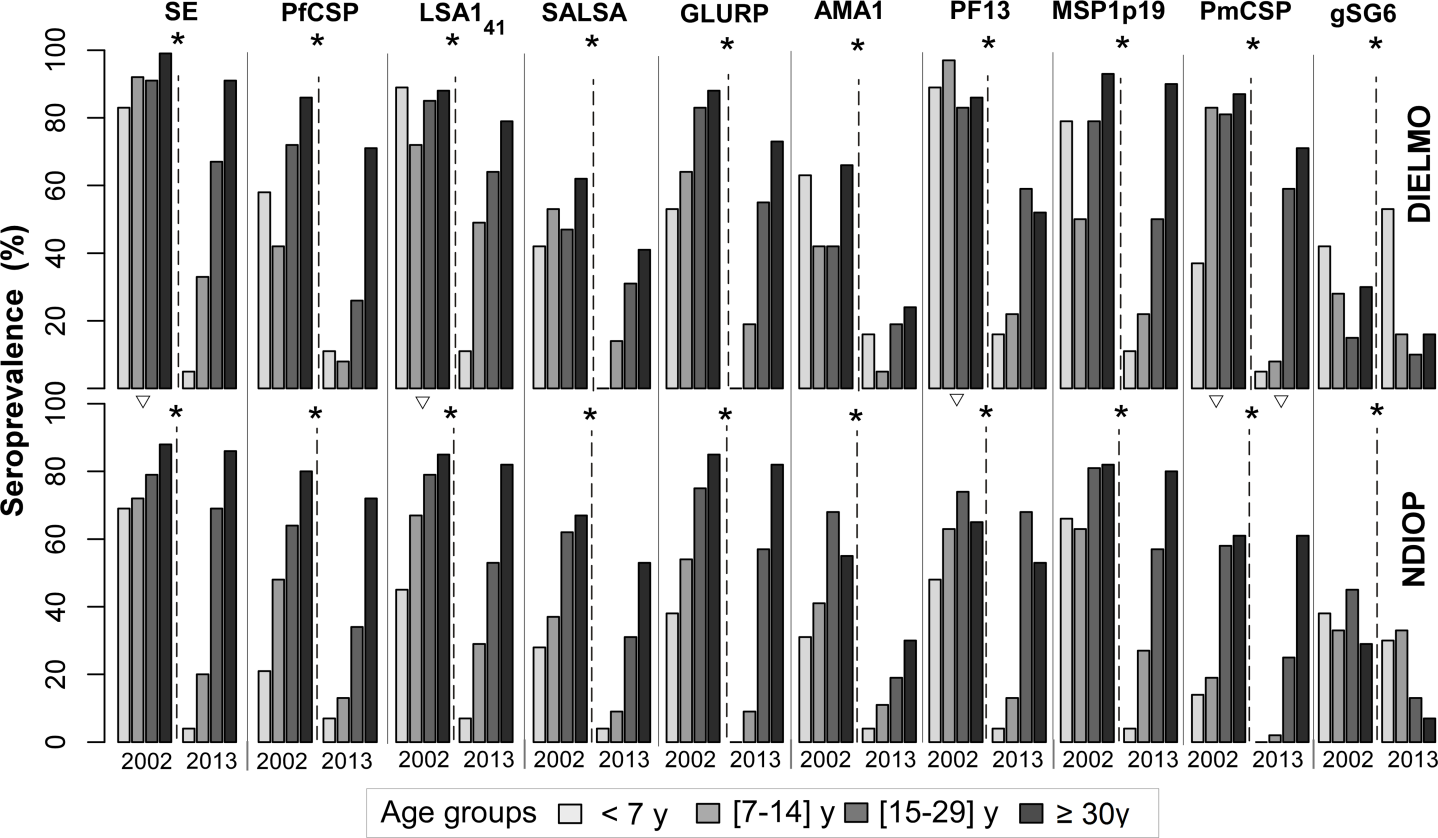
Seroprevalence observed for the panel of antigens studied during the July 2002 andJuly 2013 cross-sectional surveys conducted in Dielmo and Ndiop, by age group. Seroprevalence (percent positive individuals within each age group) to the set of antigens used are shown as bar plot for Dielmo (upper part) and Ndiop (lower part) in 2002 and 2013 for the different age groups <7y (white), 7–14 y (light grey), 15–29 y (dark grey and ≥30 y (black). Asterisks indicate a significant decrease of antibody prevalence within each community between 2002 and 2013 (P<10^−2^). Triangles indicate a significant inter-village difference of prevalence against SE, LSA1_41_, PF13, PmCSP in 2002 and PmCSP in 2013. The number of individuals within each age group for each survey and the P values of the various comparisons are presented in the S1 Table.

For both Dielmo and Ndiop, stratification by age (<7, 7–14, 15–29 and >30 years) showed quite different age-associated profiles in 2002 and 2013 (Fig 1, details in S1 Table). Seroprevalence against all antigens except gSG6 was very low (0 to <20%) in the 0-6y children in 2013. Seroprevalence against PfCSP, SALSA, GLURP, AMA1, PF13 and PmCSP was much lower in 2013 than in 2002 in the 7-14y old children. The age-associated profiles, which differed between the villages in 2002 for SE, LSA1_41_, PF13 and PmCSP (Chi-square test P<0.01 for each comparison), were similar in 2013, except for PmCSP.

### Decreased magnitude and breadth of the responses

**Fig 2:**
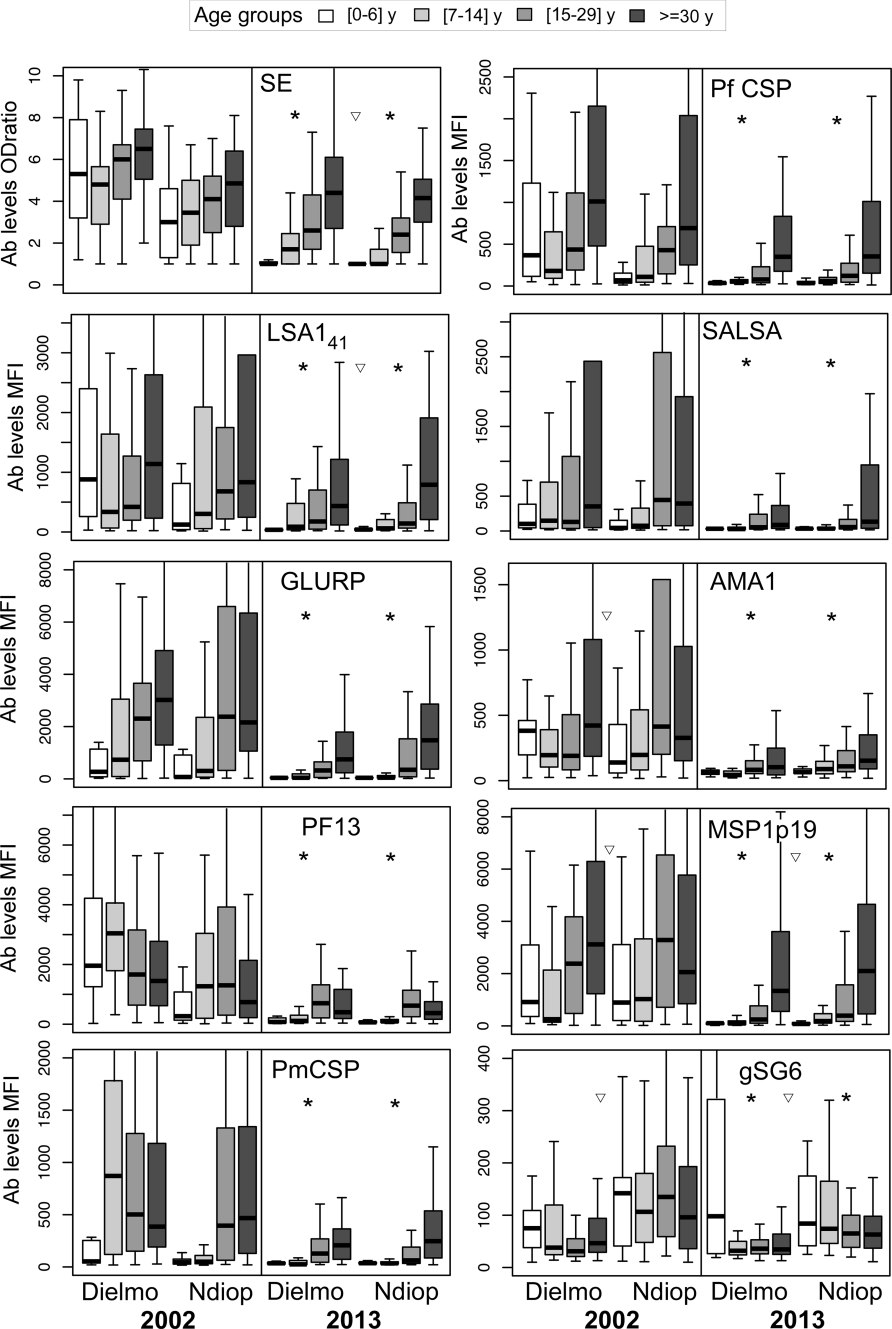
Distribution of antibody levels against ten antigens in Dielmo and Ndiop during the cross sectional surveys conducted in July 2002 and July 2013, by age group. Antibody levels against the ten antigens observed in four age groups <7y (white), 7–14 y (light grey), 15–29 y (dark grey) and ≥30 y (black) in Dielmo and Ndiop are shown as box-and-whisker plots. The box shows the first to the third quartile range, the band inside represents the median, the ends of the whiskers represent the 95% percentiles. Asterisks above the 2013 distributions indicate a significant intra-village decrease between 2002 and 2013 (P<10^−2^). Triangles indicate a significant inter-village difference of levels, *ie* against SE, AMA1, MSP1p19, gSG6 in 2002 and SE, LSA1_41_, MSP1p19, gSG6 in 2013. The number of individuals within each age group for each survey and the P values of the various comparisons are presented in the S2 Table.

In each village, the age-associated antibody levels differed between 2002 and 2013 (P<10^−3^ for all antigens, except gSG6)(Fig 2, see details S2 Table). In both villages, age was weakly correlated (correlation coefficient R = 0.30, P<10^−2^) in 2002 with antibody levels against SE, MSP1p19, GLURP, AMA1, PfCSP as well as against LSA1_41_and PmCSP in Ndiop. In2013, a stronger correlation with age was observed for all responses except against gSG6 (R = 0.55 to 0.62, depending on the antigen P<10^−2^).

The 2013 profiles were characterised by marginal IgG levels to all parasite antigens in the <6y children, low in the 7-14y children, higher in the 15-29y individuals and apart from PF13, further increased in the oldest group. Specific profiles were observed for gSG6. In Dielmo, a wide range of IgG levels was observed in the 0-6y olds and a low response in the older age groups. In Ndiop, the IgG levels against gSG6 tended to decrease with age and to be lower in 2013 compared to 2002.

The breadth of the response decreased between 2002 and 2013. The mean number of individual antigens recognised per person dropped in Dielmo from 6, 5, 6, 7 to 1, 2, 4 and 5 in the <7y, 7-14y, 15–29, >30y old groups, respectively (Mann Whitney test, P<10^-3^for all age groups) and in Ndiop from 3, 4, 6, 6 to 1, 2, 4, 5 in the <7y, 7-14y, 15–29, >30y old groups, respectively (*idem*, P<10^-3^for the first three age groups, P = 0.014for the oldest age group).

### Longitudinal analysis of antibody decay in previously immune age groups

**Fig 3:**
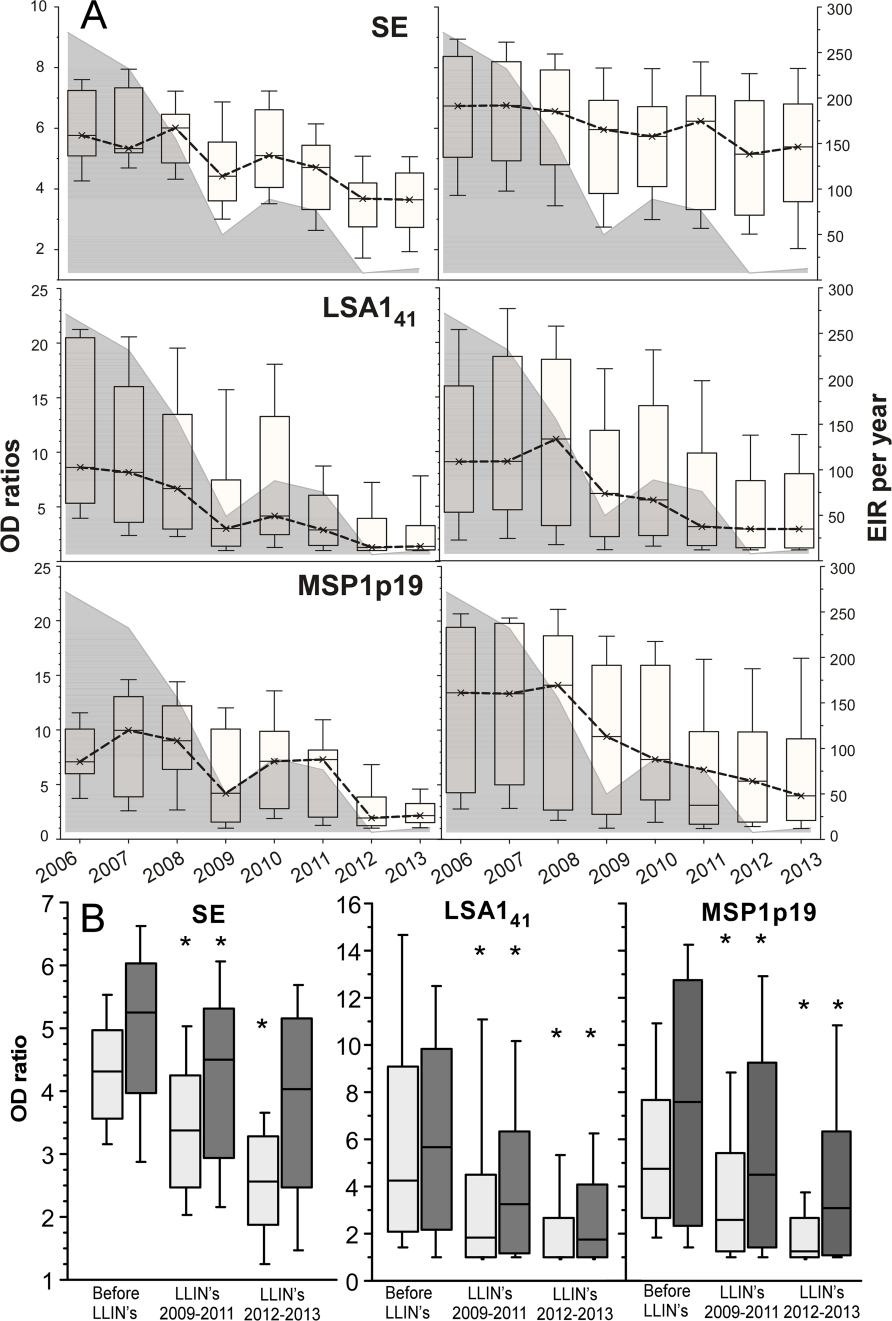
Temporal distribution of entomological inoculation rate and antibody levels to three *P*. *falciparum* antigens in two age groups from Dielmo during the 2006–2013 period. The entomological inoculation rate (EIR, the mean number of infected mosquito bites per person per year) is shown for the12 month-period extended from July1^st^ till June 30^th^ of the following year. In part (**A**), the antibody levels against SE, LSA1_41_ and MSP1p19 are shown as box-and-whisker plots of the OD ratios (left y axis) each year from 2006 to 2013 for young individuals (left part) and older individuals (right part). The box shows the first to the third quartile range, the band inside represents the median value; the limits of the whiskers represent the 95% percentiles. The temporal evolution of the median is shown by the dotted line. The temporal evolution of the EIR (right y Axis) is plotted as the background grey area. In part (**B**) antibody responses are summarized as box-and-whisker plots (as above) by age group, and before and after LLIN distribution in 2008 and 2011. Asterisks indicate significant different levels for a given age group between two successive time periods (P<10^−2^, Mann Whitney Test).

To investigate how the changes of transmission after control intensification affected the acquired antibody responses, we focused on the period from 2006 to 2013 in Dielmo. The EIR declined substantially after LLINs deployment in 2008, rebounded in 2010 and declined to low levels after new LLINs were distributed in 2011 (Fig 3). Samples from age groups that were immune in 2006, namely 17 children aged 7-14y and 47 adults aged 15–63.5 y in 2006 were analysed on a yearly basis. IgG responses against LSA1_41_, MSP1p19 as well as SE were studied in parallel assays by ELISA. ELISA was used here to homogenise the experimental procedures in this study and to provide a basis of comparison with published studies [4, 5, 9, 12, 15, 16, 19, 26–30, 32, 36, 38, 39, 52, 56, 58].

**Fig 4:**
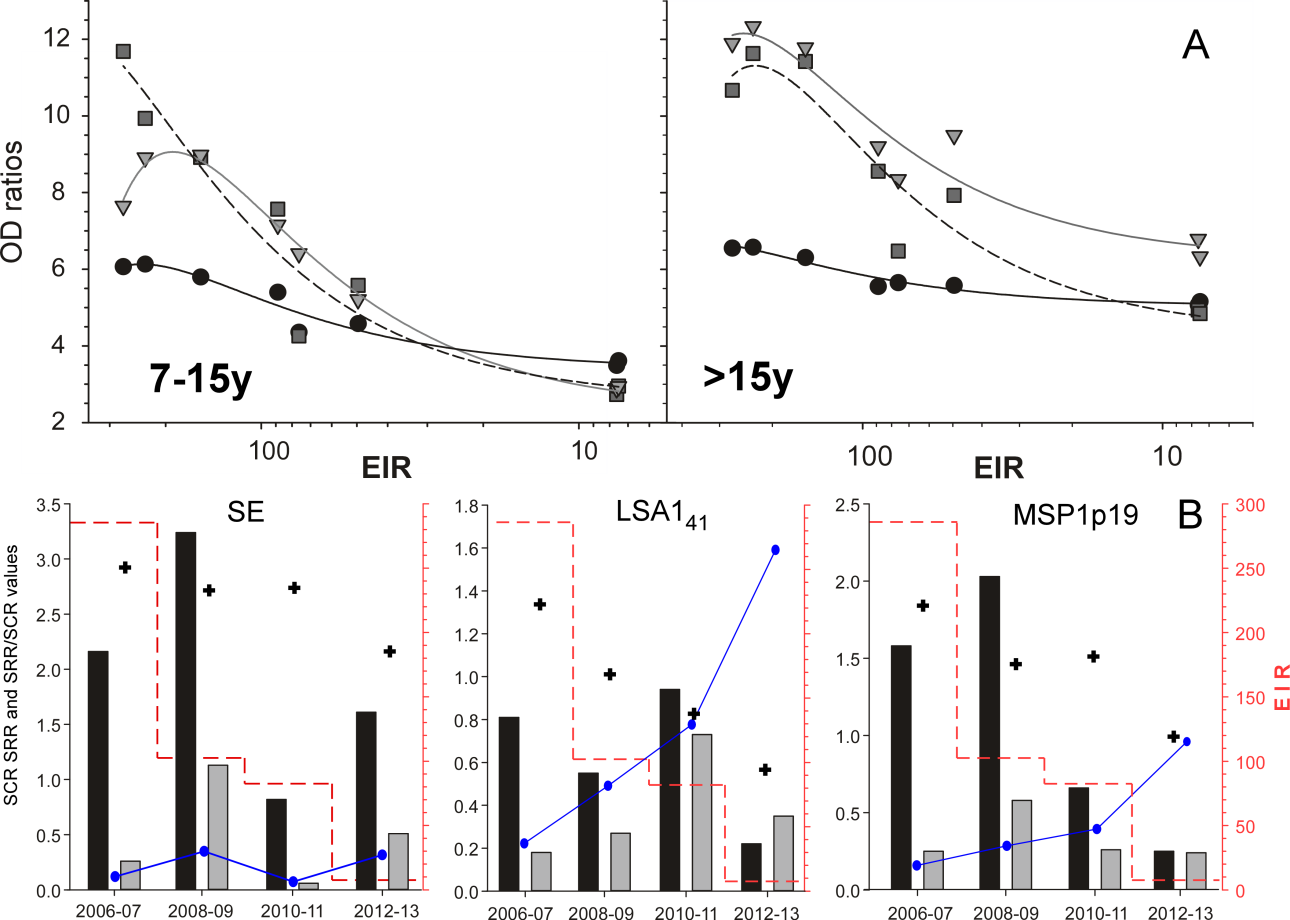
Correlation between entomological inoculation rate and mean IgG levels against three *P*. *falciparum* antigens observed in two age groups from Dielmo during the years 2006–2013. **A**. Polynomial regression curves for the relationship between the median antibody responses to SE (black circles), LSA1_41_ (grey squares), MSP1p19 (grey triangles) and EIR (log scale) for the two age groups (y = y_0_ + ax + bx^2^ + cx^3^). **B**. Seroconversion (SCR) and seroreversion (SRR) rates calculated using a 2-year stratification illustrated as bar plot for each antigen (black = SCR, grey = SRR). The temporal evolution of the SRR/SCR ratio is plotted as the plain line connecting the dot point values. The mean EIR (right y axis) for each period is plotted as the step vertical dashed red line. The black crosses indicate the mean prevalence of antibody responses for a given period (the scale used for 100% and 50% is 3.0 and 1.5, respectively on the left y axis).

Antibody levels against the three antigens fluctuated with transmission and overall declined with time (Kruskal Wallis test within each age group, P<10^−3^) (Fig 3A). The magnitude of the responses to the three antigens decreased in both age groups after each LLIN implementation (Fig 3B). There was a significant and strong (R>0.9, P<10^−3^) correlation between the EIR values and the magnitude of the response (i.e. the geometric mean of the OD ratios) against the three antigens in both age groups evidenced by polynomial regression analysis (Fig 4A). The decreases were antigen- and age-specific, more rapid and larger in children than in adults for the three antigens. The decrease of antibody levels over the 8-year period was confirmed using the Cochran-Armittage trend test, which showed a general significant decreasing trend (P<10^−4^). However, when restricted to adults, the P values were lower for MSP1p19 (P = 0.015) and met borderline significance for SE (P = 0.055). These data showed different kinetics of antibody decline in previously immune children and adults depending on the antigen. The best association with EIR was observed for the anti-MSP1p19 response in children (R = 0.99, polynomial regression, P<10^−3^).

Mathematical modelling of the seroprevalence data using an age-specific reversible catalytic conversion model [12] using a 2-year stratification combining both age groups indicated antigen-specific and period-specific seroconversion (SCR) and seroreversion (SRR) rates (Fig 4B). The individual SCRs and SRRs were unrelated to the mean EIR of the corresponding 2y period but interestingly, the SRR/SCR ratio for LSA1_41_ and for MSP1p19 increased as the mean EIR decreased.

## Discussion

The Dielmo and Ndiop cohort surveys, which uninterruptedly recorded entomological and malariometric indices using a common methodology for more than twenty years, allow informative comparisons of the effect of control intensification on the immune responses at the community level. Seroprevalence, magnitude and breadth of the responses had decreased in 2013 in all age groups and the age-associated profiles were similar in both settings for many antigens. Thus, the humoral profiling of the communities in 2013 reflected the significant progress in malaria control in the recent years resulting in low incidence of cases, uncommon asymptomatic carriage and low transmission level [5, 44, 46, 49].

The low seroprevalence and marginal levels of antibodies to parasite antigens, including SE, in the youngest age group in 2013, is in line with observations several years after control intensification in The Gambia [36, 38] and after eradication of malaria in Mauritius [35]. These children were born after introduction of ACT as first line treatment and for many of them after LLINs implementation. Their response profile reflects the cumulative effects of reduced exposure in the first years of life as they slept under nets early at night, rare occurrence of clinical malaria, which was rapidly and efficiently drug cured [46] and short half-life of the antibody response in this age group[9, 26].

The marked drop in seroprevalence, magnitude and breadth of antibody responses in the 7-15y old children between 2002 and 2013 confirms observations about the anti-MSP1p19 response in The Gambia [36, 38]. By 2013, the children 7-15y old had lost a good fraction of the responses against SE, LSA1_41_, MSP1p19, SALSA, PF13, AMA1, PfCSP and gSG6observed in the youngest age group 11 years earlier. This occurred in both settings where acquired immunity was different before control intensification, likely reflecting the short half-life of the responses and the similar exposure/ transmission conditions in the few years preceding the 2013 blood sampling. At the community level, seroprevalence, magnitude and breadth of the responses declined. This included a decreased response to MSP1p19, consistent with observations in The Gambia [38], but conflicting with the very long half-life (about 50 years) of anti-MSP1p19 antibodies estimated from comparisons of cross-sectional studies in settings with differing transmission conditions [29].

The longitudinal data provide interesting insights into the temporal decay of responses. Cases and parasite prevalence dropped in 2009 but a dramatic reduction of transmission occurred after 2011 only [46]. The responses against LSA1_41_ and MSP1p19 and to a lesser extent against SE correlated with the EIR in the preceding 12 months (Fig 4A) suggesting that they are affected by recent exposure to parasite infection and hence more labile than appreciated. Mathematical modelling showed that the SCRs and SRRs were antigen-specific and poorly related to the mean EIR in the previous 24 months, but interestingly the SRR/SCR ratio increased as the EIR collapsed. The sample size of the longitudinal study did not allow modelling the antibody decay on a yearly basis in the two age groups considered. Additional studies are needed to clarify whether the SRR/SCR ratio is age-specific as suggested by both the cross-sectional data and the relationship of antibody levels with EIR (Figs 3 and 4A).

Limitations of this study include the timing of the cross-sectional surveys. We studied the antibodies present after 5–7 months of dry season, and consequently short-lived responses and responses elicited by recent infections could remain undetected. This limitation should however have little impact in Dielmo where breeding sites and mosquitoes are present all the year [46, 48]. It would thus be interesting to investigate in Dielmo the responses to antigens identified recently as signing exposure in the past 3–6 months[24], and to carry out additional surveys at the end of the rainy season to track possible recent infections. A second limitation is the uncertainty about residual asymptomatic carriage, known to affect the immune response [3, 34]. Parasitological surveillance showed uncommon microscopy-positive smears after 2008 [46, 49], none were detected in 2013 and very few sub-microscopic infections were detected using PCR (which in our hands detects less than 1 parasite per microliter of blood). This does not exclude possible infections with lower densities or that were not detected by our snapshot samplings. However, the low responses observed in 2013 in the 7-15y old children who historically had a high rate of asymptomatic infections [59], is consistent with uncommon asymptomatic carriage in the recent years.

A limited panel of individual antigens was studied here compared to published high-throughput studies[6, 11, 13, 24]. We studied the responses to SE and to an array of individual antigens known to elicit responses reaching high seroprevalences in these settings [5, 10, 15, 16, 53] such that any decay would embrace a large dynamic range. Apart from the variant PF13 [53], the antigens used here are well conserved. Temporal variation in seroprevalence/levels against these conserved antigens is thus unlikely due to temporal genetic variation of the parasites circulating in the villages. Data regarding anti-SE responses are consistent with the observed decrease from 2000 to 2010 in the 5–19 year old villagers from Dielmo and Ndiop[5]. We show here that by 2013 the decay had occurred in all previously immune groups, including adults and importantly, that the children under 6 years were essentially seronegative against SE indicating quite limited exposure to parasites in this age group. The information gathered using SE is reminiscent of observations using parasite-based assays such as immunofluorescence[35] or indirect hemagglutination [7] assays. SE has the advantage of containing a broad panel of parasite antigens, which moreover are presented in their native conformation and native post-translational modifications. The high historical seroprevalence and levels observed in both villages is consistent with previous studies [5, 56] and with the high responses observed against the various erythrocytic antigens studied here but contrasts with the low figures reported in a Kenyan setting [22], possibly reflecting differences in extract preparation. The sensitive SE-assay provides a rapid mean to monitor the anti-parasite-response, but the exact specificity of the observed signals is unknown, precluding identification of specific conversion and reversion events.

The 2002 survey confirms the high historical seroprevalence against the recombinant antigens and peptides used here [10, 15, 16, 53, 55]. All responses apart from gSG6 had declined by 2013. The response against PmCSP followed a pattern similar to PfCSP, consistent with the marked drop in *P*. *malariae* cases and infections after control scale-up [47].

The response against gSG6 presented specific age-stratified patterns and temporal trends. The peptide allows to capture of antibodies elicited by *An*. *gambiae*[17] as well as *An*. *funestus*[60], which are the main vectors in Dielmo and Ndiop[43, 46]. The lower community seroprevalence in 2013 compared to 2002 is consistent with the published association with transmission intensity [27, 61, 62]. The lower responses in the older age groups observed in both villages in 2013 are in line with the reported tolerance response in the older subjects [33, 63, 64]. The large heterogeneity of antibody levels observed in the young children in Dielmo in 2013 is surprising, as antibodies to gSG6 have been reported to be negatively associated with the use of LLIns in young children [65]. It contrasts with the more homogeneous response observed in the same age group 11 years earlier and suggests substantial heterogeneity of exposure of young children to *Anopheles* bites in 2013, as reported in low transmission conditions [62]. Presence of antibodies against gSG6 indicates continued exposure to mosquito bites in 2013 is consistent with the recorded vector density in the villages, and probably indicates exposure to *Anopheline* bites at a time when children are not using LLINs. Indeed, the low/nil responses of these young children against parasite antigens reflect the combined benefit of limited/nil exposure to infectious bites thanks to LLINs and limited exposure to parasites thanks to the use of efficacious antimalarial treatments.

The panel of antigens analysed here provided a consistent picture of declining immunity and allowed to identify clear signatures of progress towards malaria elimination. The list of potential biomarkers is not closed and could obviously be completed with additional antigens identified recently in other settings as associated with recent infections [24]. Our data illustrate the value of community surveys for documenting malaria decline. A few consecutive years of reduced exposure decreased specific immunity against pre-erythrocytic and blood stage antigens whatever the pre-existing level of immunity in the community. The young children born after control intensification had quite limited if any acquired immunity against parasite antigens. This age group is particularly vulnerable to possible failures in control and highly informative in case of such circumstances, but is poorly informative for monitoring further progress towards elimination. The older children have lost a good fraction of their pre-existing immunity as transmission declined. There was clear evidence of waning immunity in adults, with declining antibody levels but still substantial seroprevalence levels, consistent with the residual local transmission and the high antibody levels before control intensification. The previously immune age groups thus appear sensitive indicators of declining transmission and it will be important to continue monitoring in the future years to study how their immune responses evolve as transmission further declines.

## Supporting information

S1 Table Mean antibody prevalence against ten antigens in four age groups in Dielmo and Ndiop during the cross-sectional surveys conducted in July 2002 and July 2013. (DOCX)

S2 Table Age-associated distribution of antibody levels against ten antigens observed during the cross sectional surveys conducted in Dielmo and Ndiop in July 2002 and July 2013. (DOCX)
