# Supplementary material for: Serological signatures of declining exposure following intensification of integrated malaria control in two rural Senegalese communities
Source: PLoS One. 2017 Jun 13;12(6):e0179146. doi: 10.1371/journal.pone.0179146 (PMC5469466; doi:10.1371/journal.pone.0179146)
Supplement: S1 Table — (DOCX) [file pone.0179146.s001.docx]

**S Table1** Mean antibody prevalence against ten antigens in four age groups in Dielmo and Ndiop during the cross-sectional surveys conducted in July 2002 and July 2013

|  | **Schizont extract** | | | **PfCSP** | | | **LSA1_41_** | | | **SALSA** | | | **GLURP** | | | |
| --- | --- | --- | --- | --- | --- | --- | --- | --- | --- | --- | --- | --- | --- | --- | --- | --- |
|  | 2002 | 2013 |  | 2002 | 2013 |  | 2002 | 2013 |  | 2002 | 2013 |  | 2002 |  | 2013 |  |
| **DIELMO (No in 2002, 2013)** | % | % | *P*^a^ | % | % | *P*^a^ | % | % | *P*^a^ | % | % | *P*^a^ | % |  | % | *P*^a^ |
| < 7yrs (19, 19) | 83.3 | 5.3 | <10^-3^ | 57.9 | 10.5 | <10^-3^ | 89.5 | 10.5 | <10^-3^ | 42.1 | 0.0 | <10^-3^ | 52.6 |  | 0.0 | <10^-3^ |
| [7-14]yrs (36, 37) | 91.6 | 33.3 |  | 41.7 | 8.1 |  | 72.2 | 48.6 |  | 52.8 | 13.5 |  | 63.9 |  | 18.9 |  |
| [15-29]yrs (53, 58) | 90.6 | 67.2 |  | 71.7 | 25.9 |  | 84.9 | 63.8 |  | 47.2 | 31.0 |  | 83.0 |  | 55.2 |  |
| ≥ 30yrs (76, 82) | 98.7 | 91.3 |  | 85.5 | 70.7 |  | 88.2 | 79.3 |  | 61.8 | 41.5 |  | 88.2 |  | 73.2 |  |
| **NDIOP (No in 2002, 2013)** |  |  |  |  |  |  |  |  |  |  |  |  |  |  |  |  |
| < 7yrs (19, 19) | 69.0 | 3.7 | <10^-3^ | 20.7 | 7.4 | <10^-3^ | 44.8 | 7.4 | <10^-3^ | 27.6 | 3.7 | <10^-3^ | 37.9 |  | 0.0 | <10^-3^ |
| [7-14]yrs (36, 37) | 72.2 | 20.0 |  | 48.1 | 13.3 |  | 66.7 | 28.9 |  | 37.0 | 8.9 |  | 53.7 |  | 8.9 |  |
| [15-29]yrs (53, 58) | 79.2 | 68.7 |  | 64.2 | 33.8 |  | 79.2 | 52.9 |  | 62.3 | 30.9 |  | 75.5 |  | 57.4 |  |
| ≥ 30yrs (76, 82) | 87.9 | 85.5 |  | 80.3 | 72.4 |  | 84.8 | 81.6 |  | 66.7 | 52.6 |  | 84.8 |  | 81.6 |  |
| **Inter-village comparison** | |  |  |  |  |  |  |  |  |  |  |  |  |  |  |  |
| P-value^b^ | <10^-3^ | 0.27 |  | 0.096 | 0.26 |  | 0.016 | 0.13 |  | 0.94 | 0.39 |  | 0.06 |  | 0.62 |  |
|  | **AMA1** | | | **PF13** | | | **MSP1p19** | | | **PmCSP** | | | **gSG6** | | | |
|  | 2002 | 2013 |  | 2002 | 2013 |  | 2002 | 2013 |  | 2002 | 2013 |  | 2002 |  | 2013 |  |
| **DIELMO (No in 2002, 2013)** | % | % | *P*^a^ | % | % | *P*^a^ | % | % | *P*^a^ | % | % | *P*^a^ | % |  | % | *P*^a^ |
| < 7yrs (19, 19) | 63.2 | 15.8 | <10^-3^ | 89.5 | 15.8 | <10^-3^ | 78.9 | 10.5 | <10^-3^ | 36.8 | 5.3 | <10^-3^ | 42.1 |  | 52.6 | 0.04 |
| [7-14]yrs (36, 37) | 41.7 | 5.4 |  | 97.2 | 21.6 |  | 50.0 | 21.6 |  | 83.3 | 8.1 |  | 27.8 |  | 16.2 |  |
| [15-29]yrs (53, 58) | 41.5 | 19.0 |  | 83.0 | 58.6 |  | 79.2 | 50.0 |  | 81.1 | 58.6 |  | 15.1 |  | 10.3 |  |
| ≥ 30yrs (76, 82) | 65.8 | 24.4 |  | 85.5 | 52.4 |  | 93.4 | 90.2 |  | 86.8 | 70.7 |  | 30.3 |  | 15.9 |  |
| **NDIOP (No in 2002, 2013)** |  |  |  |  |  |  |  |  |  |  |  |  |  |  |  |  |
| < 7yrs (19, 19) | 31.0 | 3.7 | <10^-3^ | 48.3 | 3.7 | <10^-3^ | 65.5 | 3.7 | <10^-3^ | 13.8 | 0.0 | 1.4x10^-3^ | 37.9 |  | 29.6 | <10^-3^ |
| [7-14]yrs (36, 37) | 40.7 | 11.1 |  | 63.0 | 13.3 |  | 63.0 | 26.7 |  | 18.5 | 2.2 |  | 33.3 |  | 33.3 |  |
| [15-29]yrs (53, 58) | 67.9 | 19.1 |  | 73.6 | 67.6 |  | 81.1 | 57.4 |  | 58.5 | 25.0 |  | 45.3 |  | 13.2 |  |
| ≥ 30yrs (76, 82) | 54.5 | 30.3 |  | 65.2 | 52.6 |  | 81.8 | 80.3 |  | 60.6 | 60.5 |  | 28.8 |  | 6.6 |  |
| **Inter-village comparison** | |  |  |  |  |  |  |  |  |  |  |  |  |  |  |  |
| P-value^b^ | 0.81 | 0.54 |  | <10^-3^ | 0.98 |  | 0.44 | 0.81 |  | <10^-3^ | <10^-3^ |  | 0.06 |  | 0.64 |  |
|  |  |  |  |  |  |  |  |  |  |  |  |  |  |  |  |  |
| ^a^ comparison between years 2002 and 2013 ajusted on age, in each village (logistic regression) | | | | | | | | |  |  |  |  |  |  |  |  |
| ^b^ intervillage comparison of each survey ajusted on age (logistic regression) | | | | | | |  |  |  |  |  |  |  |  |  |  |
