# Supplementary material for: Serological signatures of declining exposure following intensification of integrated malaria control in two rural Senegalese communities
Source: PLoS One. 2017 Jun 13;12(6):e0179146. doi: 10.1371/journal.pone.0179146 (PMC5469466; doi:10.1371/journal.pone.0179146)
Supplement: S2 Table — (DOCX) [file pone.0179146.s002.docx]

|  |  | **Schizont extract** | | |  | **PfCSP** | | |  | **LSA1_41_** | | |  | **SALSA** | | |  | **GLURP** | | |
| --- | --- | --- | --- | --- | --- | --- | --- | --- | --- | --- | --- | --- | --- | --- | --- | --- | --- | --- | --- | --- |
|  |  | OD ratio^a^ | |  |  | MFI | |  |  | MFI | |  |  | MFI | |  |  | MFI | |  |
| **DIELMO (No in 2002, 2013)** | | 2002 | 2013 | *P*^c^ |  | 2002 | 2013 | *P*^c^ |  | 2002 | 2013 | *P*^c^ |  | 2002 | 2013 | *P*^c^ |  | 2002 | 2013 | *P*^c^ |
| *P^b^* |  | <10^-2^ | <10^-2^ |  |  | <10^-2^ | <10^-2^ |  |  | 0.05 | <10^-2^ |  |  | 0.2 | <10^-2^ |  |  | <10^-2^ | <10^-2^ |  |
| < 7yrs (19, 19) |  | 5.3 | 1.2 | <10^-3^ |  | 1170 | 263 | <10^-3^ |  | 2372 | 176 | <10^-3^ |  | 471 | 35 | <10^-3^ |  | 1464 | 54 | <10^-3^ |
| [7-14]yrs (36, 37) |  | 4.4 | 2.1 |  |  | 320 | 88 |  |  | 1048 | 388 |  |  | 920 | 125 |  |  | 1940 | 196 |  |
| [15-29]yrs (53, 58) |  | 5.6 | 3.0 |  |  | 681 | 254 |  |  | 1186 | 581 |  |  | 869 | 224 |  |  | 2576 | 572 |  |
| ≥ 30yrs (76, 82) |  | 6.3 | 4.6 |  |  | 1663 | 728 |  |  | 2178 | 1081 |  |  | 1604 | 553 |  |  | 3433 | 1345 |  |
| **NDIOP (No in 2002, 2013)** |  |  |  |  |  |  |  |  |  |  |  |  |  |  |  |  |  |  |  |  |
| *P^b^* |  | <10^-2^ | <10^-2^ |  |  | <10^-2^ | <10^-2^ |  |  | <10^-2^ | <10^-2^ |  |  | <10^-2^ | <10^-2^ |  |  | <10^-2^ | <10^-2^ |  |
| < 7yrs (19, 19) |  | 3.2 | 1.2 | <10^-3^ |  | 247 | 79 | <10^-3^ |  | 930 | 67 | <10^-3^ |  | 364 | 45 | <10^-3^ |  | 975 | 47 | <10^-3^ |
| [7-14]yrs (36, 37) |  | 3.5 | 1.7 |  |  | 502 | 216 |  |  | 1679 | 790 |  |  | 1037 | 56 |  |  | 2523 | 199 |  |
| [15-29]yrs (53, 58) |  | 3.9 | 2.5 |  |  | 739 | 233 |  |  | 2118 | 604 |  |  | 2183 | 388 |  |  | 3731 | 1179 |  |
| ≥ 30yrs (76, 82) |  | 4.7 | 4.0 |  |  | 1513 | 822 |  |  | 2265 | 1873 |  |  | 1605 | 776 |  |  | 3941 | 2272 |  |
| **Inter-village comparison** *P^d^* |  | <0.01 | <10^-2^ |  |  | 0.5 | 0.64 |  |  | 0.35 | 0.03 |  |  | 0.12 | 0.15 |  |  | 0.09 | 0.09 |  |
|  |  |  |  |  |  |  |  |  |  |  |  |  |  |  |  |  |  |  |  |  |
|  |  | **AMA1** | | |  | **PF13** | | |  |  | **MSP1p19** |  |  | **PmCSP** | | |  |  | **gSG6** | |
|  |  | MFI | |  |  | MFI | |  |  | MFI | |  |  | MFI | |  |  | MFI | |  |
| **DIELMO (No in 2002, 2013)** | | 2002 | 2013 | *P*^c^ |  | 2002 | 2013 | *P*^c^ |  | 2002 | 2013 | *P*^c^ |  | 2002 | 2013 | *P*^c^ |  | 2002 | 2013 | *P*^c^ |
| *P^b^* |  | <10^-2^ | <10^-2^ |  |  | <10^-2^ | <10^-2^ |  |  | <10^-2^ | <10^-2^ |  |  | <10^-2^ | <10^-2^ |  |  | 0.02 | 0.13 |  |
| < 7yrs (19, 19) |  | 425 | 101 | <10^-3^ |  | 2636 | 204 | <10^-3^ |  | 1754 | 355 | <10^-3^ |  | 614 | 40 | <10^-3^ |  | 91 | 200 | 0.05 |
| [7-14]yrs (36, 37) |  | 306 | 88 |  |  | 3363 | 247 |  |  | 1488 | 366 |  |  | 1442 | 79 |  |  | 86 | 52 |  |
| [15-29]yrs (53, 58) |  | 383 | 149 |  |  | 1997 | 919 |  |  | 2637 | 627 |  |  | 987 | 311 |  |  | 63 | 72 |  |
| ≥ 30yrs (76, 82) |  | 1009 | 277 |  |  | 1997 | 765 |  |  | 4069 | 2575 |  |  | 1058 | 317 |  |  | 116 | 59 |  |
| **NDIOP (No in 2002, 2013)** |  |  |  |  |  |  |  |  |  |  |  |  |  |  |  |  |  |  |  |  |
| *P^b^* |  | <10^-2^ | <10^-2^ |  |  | 0,03 | <10^-2^ |  |  | 0.01 | <10^-2^ |  |  | <10^-2^ | <10^-2^ |  |  | 0.23 | 0.05 |  |
| < 7yrs (19, 19) |  | 343 | 81 | <10^-3^ |  | 1297 | 98 | <10^-3^ |  | 2574 | 185 | <10^-3^ |  | 546 | 41 | <10^-3^ |  | 176 | 160 | 0.01 |
| [7-14]yrs (36, 37) |  | 798 | 150 |  |  | 2180 | 164 |  |  | 2631 | 550 |  |  | 786 | 49 |  |  | 152 | 171 |  |
| [15-29]yrs (53, 58) |  | 1200 | 246 |  |  | 2441 | 867 |  |  | 4148 | 1409 |  |  | 1635 | 166 |  |  | 318 | 109 |  |
| ≥ 30yrs (76, 82) |  | 947 | 358 |  |  | 1629 | 685 |  |  | 3913 | 3261 |  |  | 1534 | 733 |  |  | 182 | 107 |  |
| **Inter-village comparison** *P^d^* |  | 0.03 | 0.08 |  |  | 0.06 | 0.34 |  |  | 0.04 | 0.02 |  |  | 0.38 | 0.21 |  |  | <0.01 | 0.01 |  |
|  |  |  |  |  |  |  |  |  |  |  |  |  |  |  |  |  |  |  |  |  |
| ^a^ Mean IgG levels expressed in OD ratio (schizont extract) or MFI for defined antigens | | | | | | | | | | | |  |  |  |  |  |  |  |  |  |
| ^b^ Variation of IgG levels between the various age-groups at each survey (year 2002, year 2013) ( Kruskal-Wallis test) | | | | | | | | | | | | | | | | | |  |  |  |
| ^c^ Comparison of IgG levels between 2002 and 2013 adjusted for age group in the village (Kruskal-Wallis test) | | | | | | | | | | | | | | | | |  |  |  |  |
| ^d^ Between-village comparison of values for each cross sectional survey, adjusted on age groups | | | | | | | | | | | | | |  |  |  |  |  |  |  |
